# Health Tract, No. 133

**Published:** 1863-04

**Authors:** 


HEALTH TRACT, No. 133.

### LIFE WASTED.

VALUABLE lives are often thrown away, lost, through ignorance of some of the simplest truths in nature, or errors of judgment in matters where error becomes a crime. Some of the best and wisest and greatest men of our race have perished from the world, in consequence of what might be considered a carelessness, a recklessness, or an ignorance, which is amazing, as found in minds like theirs. The immediate cause of Lord Bacon's death was sleeping in a damp bed. Any old woman, who "didn't know B from Bull's foot," would have had more sense than that. Yet it was the fatal error of the greatest mind of his age and generation.

Washington Irving, whose name is so loved and honored and revered, hastened his death by taking the advice of a fool, instead of his physician. Abbott Lawrence, the financier and the philanthropist, brought on his last illness by an injudicious change of clothing.

Rachel, the greatest tragic actress of her time, took a cold which carried her to her grave, by riding from New-York to Boston in cars not sufficiently warmed, on a bitter cold winter's night, immediately after a performance, which had heated up her whole system, far beyond its natural standard. J. Addison Alexander, for whom it is claimed that he was the best Bible scholar living, and that he had powers of mind not equaled in his day, died in the very prime of life, because "having a feeling almost bordering on contempt for physicians," he allowed his mortal malady to prey upon him secretly; and the day he died, he thought he was going to get well. Because he knew nothing about disease, he concluded, with all his resplendent intellect, that men who had made it a life-long study, knew nothing about it. The magnificent deduction cost him his life. And now another name comes up to our notice, in the same connection, as illustrating the fact that the greatest minds are capable of follies most amazing. The philosopher, the scholar, the soldier, and the Christian, were all blended in the name of Professor Mitchel, the great astronomer, the gallant soldier, and resistless general. His was the greatest loss to the nation, up to this hour of the contest, and yet his life was literally thrown away, by his own inconsiderate act; by doing deliberately what we would suppose the commonest mind in the nation would have regarded as exceedingly dangerous; and it is named here to benefit the living, without prejudice to the honored and lamented dead. General Mitchel was attacked with symptoms of yellow fever; his physician acted promptly, and labored to restore the functions of the skin, to cause perspiration, which every professional man knows is the turning-point for life in that disease. It was eventually brought about, to the unspeakable joy of his medical attendant, Dr. Thomas T. Smiley, at twelve M., October 28th, 1862; but when he returned, two hours later, his patient had been attacked with a chill, the pulse went up from 85 to 120, the General having got up and ordered his bed changed, *while in this perspiring condition.* Delirium set in, and he died, the attending surgeon leaving this record: "I am of the opinion that had General Mitchel remained in bed, and kept the skin in good condition, he would without doubt have recovered."

